# Supplementary material for: Are carbon emissions trading and green financial instruments synergistic? -Comprehensive quantitative research based on content analysis
Source: PLoS One. 2024 Mar 7;19(3):e0298601. doi: 10.1371/journal.pone.0298601 (PMC10919601; doi:10.1371/journal.pone.0298601)
Supplement: S1 Data — (ZIP) [file pone.0298601.s001.zip › Data/地级市Z_F工作报告.zip!/─┌├╔╣┼/║⌠┬╫▒┤╢√╩╨/└┤╫╘╣1⁄2╓┌║┼í╛┬φ┐╦╩2╛▌═°í┐_2007.docx]

各位代表：

现在，我代表呼伦贝尔市人民政府向大会作工作报告，请予审议，并请市政协委员和列席人员提出意见。

一、2006年工作回顾

2006年，是“十一五”的开局之年。在自治区党委、政府和市委的正确领导下，我们以科学发展观为指导，解放思想，真抓实干，经济社会呈现出协调并进、蓄势聚能、快速发展的良好态势。全市地区生产总值完成395.06亿元，继续保持了两位数的高位增长，增速达到18%。人均地区生产总值完成14621元，增长18％。固定资产投资完成180.05亿元，增长2.8%。财政总收入完成49.86亿元，增长39.59％，其中地方财政收入完成29.1亿元，增长30.89％。城镇居民人均可支配收入完成9051元，增长10％。农牧民人均纯收入达到3608元，增长12.7%。社会消费品零售总额达到126.45亿元，增长16.9％。旅游业已成为新的经济增长点，总收入完成53.8亿元，增长16.7%。我市荣获了CCTV2006年度“中国最佳民族风情魅力城市”称号，满洲里市荣获了CCTV2006年度“中国十佳魅力城市”称号，使我市的知名度迅速攀升。

工业经济质量效益同步提高。全市规模以上工业增加值完成84.89亿元，增长30.2%。实施工业重点项目86个，完成工业固定资产投资89.44亿元，规模以上企业实现利润28.5亿元，增长106.2%。扎兰屯百业城百万吨玉米酒精、大庆石油开发、陈旗宝日希勒千万吨露天矿、新右旗国华风电5万千瓦风力发电等一批重点项目竣工，部分项目已经投产。伊敏二期、五牧场长焰煤基地、满洲里三发木材加工、雀巢集团大型乳粉加工、海拉尔农垦油菜籽产业化等重点项目开工建设。360万千瓦外送电力、80万千瓦热电联产、上海重工40万吨甲醇、牙克石新大洲电石法PVC等项目前期工作进展顺利，博世集团冬季汽车性能测试场项目顺利实施。瑞士雀巢、德国博世集团的入驻，开创了国际化世界500强入驻内蒙古的先例。

新农村新牧区建设稳步推进。认真贯彻落实中央2006年1号文件精神，投放支农支牧资金 6.72亿元，增长33.2%。农牧林区经济稳步发展，粮食产量达到61亿斤，牧业年度牲畜存栏达到1538.4万头（只）；林区绿色食品加工和采集业快速发展，地方林业加工山野菜7312吨，实现销售收入9648万元。重点推进了新农村新牧区试点建设，制定了《新农村牧区建设五年规划》，完成了13个重点嘎查村规划。加大了扶贫工作力度，投入1770万元，在59个嘎查村实施了整村推进工程。积极改善农牧林区基础设施条件，全市节水灌溉面积达到25.87万亩，新建通乡公路328公里、通村公路235公里。继续实施广播电视村村通工程，广播电视人口覆盖率达到85.5%。农村牧区教育、医疗卫生等事业得到进一步加强。积极实施“兴边富民行动”，市本级安排少数民族新民居建设补贴及民族旗市补助100万元，争取自治区各类专项资金6545万元，重点扶持了鄂温克、鄂伦春、达斡尔和俄罗斯等少数民族地区经济社会发展。新农村新牧区建设实现了良好开局。

生态和基础设施建设得到加强。草原建设总规模完成1431万亩，退耕106万亩，禁牧休牧4187万亩。重点公益林保护面积830万亩。尤其是森工集团坚持生态优先的原则，强化森林管护措施，有林地面积增加2.4万公顷，森林覆盖率达到了76.8%,提高了0.23个百分点，活立木蓄积量达到7.18亿立方米，增加了410万立方米。水利建设完成投资11.7亿元，尼尔基水利枢纽主体工程全部竣工并已上网发电，组织实施了农田草牧场节水灌溉、水土保持、黑土地治理、安全饮水、水库除险加固、防洪与界河治理等项目。城市基础设施建设完成投资11.15亿元，城关镇、中心城镇给排水、垃圾处理、城镇道路改造和园林绿化等项目建设步伐明显加快，海拉尔区垃圾处理工程和三角地地下人防工程等一大批城镇基础设施建设项目竣工并投入使用；市新城区建设完成投资10亿元，在6.5平方公里的起步区，完成了新区市政基础设施建设一期工程、“六横五纵”22条主次干道、支路路网工程和240公里的管网工程，新城区已初具雏形。满洲里口岸国际大酒店、通联大厦等欧式高层建筑进一步提升了边城的品位。公路建设完成投资25.07亿元，301线海拉尔至满洲里190公里一级公路、国道111线大杨树至白桦排66公里二级公路等11项建设工程顺利实施，公路等级有了大幅度提升。“两伊”铁路和滨洲铁路海满段复线建设全面推进。民航海拉尔新航站区投入使用，开工建设了满洲里航站改（扩）建项目。

改革开放取得新进展。国有大企业改制重组、优化整合力度加大，投资主体多元化步伐加快，神华集团整体接收宝煤公司，鲁能集团与雁煤公司实现资产组合，华能集团整体重组并购了扎煤公司。农村牧区综合改革扎实推进，全部免除了农牧业税费。苏木乡镇机构改革全部完成，精简比例达到40％。农垦企业进行了集团化、股份化改革。财税、金融、流通等领域改革都取得了积极进展。对外开放迈出新步伐，全年实施招商引资项目648项，累计引进市外（国内）资金151.7亿元，增长43.9%。成功举办了中俄蒙边境区域合作论坛及中俄蒙毗邻地区矿产资源、房地产开发和农业合作项目对接会。大庆石油公司、新鑫矿业公司开发蒙古国塔木察格油田、乌兰铅锌矿取得新进展。海拉尔至俄罗斯赤塔国际航线由包机飞行转为正式国际航班，阿日哈沙特口岸联检楼投入使用，额布都格至蒙古国白音胡舒口岸大桥、黑山头至俄罗斯旧粗鲁海图口岸大桥竣工通车。边检、海关、出入境检验检疫、铁路等联检联运部门积极配合，有力支持了地方经济发展。全年对外贸易进出口总额完成21.29亿美元，增长33%；实际利用外资3661万美元，增长52 %；口岸进出境货运量达到2183万吨，增长23%。

人民群众得到更多实惠。落实生产资料、粮食、种子、农机、奶牛等各项惠农补贴资金1.75亿元。19.3万低保人口人均月补助水平达到87.1元，提高23.1元。建立了大学生救助长效机制，1257名贫困新生得到了一次性救助，5526名在读贫困大学生得到了全额低保救助。城镇新增就业2.76万人，城镇登记失业率控制在了4.23%。养老、失业、医疗、工伤、生育保险逐步规范，各项社会保险的覆盖面进一步扩大。贫困地区人民群众生产生活条件得到改善，解决了10万贫困人口的温饱问题。

各项社会事业全面进步。大力实施科教兴市战略，科技成果向现实生产力转化步伐加快。各级各类教育事业稳步发展，基础设施和办学条件不断改善，“两基”达标攻坚任务圆满完成，“普九”人口覆盖率达到100%。公共卫生设施建设得到加强，疾病控制、医疗救治和食品、药品、卫生监督体系逐步完善。人口和计划生育工作扎实推进，在全区获优秀奖。积极开展民族文化大市建设，文化艺术、新闻出版、社会科学事业得到加强。广播电视、体育事业快速发展，成功举办了2006中国·呼伦贝尔国际汽车越野赛暨媒体探路活动。认真贯彻落实民族宗教政策，以“三少”民族为主的少数民族地区群众生产生活条件得到明显改善。人事人才工作得到加强，完成了全市党政机关公务员登记工作，实施了全市机关事业单位工资制度改革。统计、国土、侨务、人防、地震、气象、保险等各项事业都取得了新进展。

精神文明和民主法制建设得到加强。精神文明建设取得丰硕成果，城乡居民道德素质和文明程度有了新的提升。扩大政务公开范围，推进了政府决策的科学化、民主化进程。自觉接受人大依法监督和政协民主监督，全年共办理人大代表建议、议案和政协委员提案227件。进一步加强了与各民主党派、工商联和无党派人士的联系。审计监督力度进一步加大，廉政建设和反腐败斗争不断加强。深入开展 “集中整治”和“大接访”活动，妥善处理了群众反映强烈的突出问题，较好地控制了越级上访事件和群体性事件。安全生产工作得到全面加强，控制性指标完成情况位居全区首位。积极开展普法教育，强化社会治安综合治理，严厉打击各种刑事犯罪，有力地维护了社会稳定。积极开展国防教育和国防后备力量建设，边防一体化建设扎实推进，顺利通过自治区“双拥模范城”检查验收。

为民办实事项目全部兑现。在市一届六次人代会上，市委、市政府向人民承诺的办实事项目全部兑现。一是农村牧区饮水安全工程开工建设16处，打机电井21眼，解决了3.2万人、4.15万头（只）牲畜饮水安全问题。二是贫困家庭寄宿生生活费补助面达到全市农村牧区寄宿生总数的40%，发放补助金631万元。享受民族学校少数民族贫困家庭住宿生助学金的范围达到了50%，发放助学金160 万元。三是牧区初中由24所调整为17所、小学由53所调整为25所，彻底实行了“两主一公”的办学模式。四是开工建设了建筑面积为2.1万平方米的呼伦贝尔市体育馆，海拉尔、满洲里、牙克石、阿荣旗、新右旗等地都自行安排了体育基础设施建设项目。五是改造街巷道路136条，总面积61.5万平方米，新装路灯1600盏，居民的出行环境得到改善。六是实施了“蓝天工程”，进一步解决了冬季煤烟大气污染问题。

各位代表，在过去的一年里，全市各族人民团结拼搏、知难而进，取得了经济快速发展、社会全面进步的可喜成就。这是自治区党委、政府正确领导和亲切关怀的结果，是市委科学决策、市人大依法监督、市政协民主监督的结果，是各级、各部门密切协作、优化环境、真抓实干的结果，是全市各族人民共同努力和社会各界大力支持的结果。在此，我代表市人民政府，向奋战在各条战线上的广大干部群众表示亲切的慰问！向驻市部队、武警官兵和公安干警致以崇高的敬意！向全力支持政府工作的各位代表、各位委员和社会各界人士表示衷心的感谢！

在充分肯定成绩的同时，我们也清醒地认识到，前进中还存在不少困难和问题。主要有：经济社会发展总体水平比较低，尤其是经济总量小，与西部发展较快盟市还存在一定差距；工业经济缺乏大项目支撑，原字号、初级产品多，科技含量和附加值不高，对一产和三产的拉动力不强；民营经济发展速度不快，培育力度不够，对全市经济发展贡献率不高；区域经济发展不平衡，各旗市区之间差距较大；基础设施建设滞后，农牧业靠天吃饭的局面没有得到根本改善，城市功能不够完善，管理水平有待进一步提高；招商引资环境需进一步优化；就业和再就业压力较大，农村牧区社会事业发展相对滞后，城乡居民收入水平不高，一些群众生活还很困难；部分干部的思想解放程度不高、创新能力不强、抓落实的力度不够。针对上述问题，我们必须高度重视，用改革的措施、发展的办法，认真加以解决。

二、2007年发展目标和任务

2007年政府工作的总体要求是：以科学发展观为指导，认真贯彻自治区第八次党代会、十届五次人代会和市二次党代会精神，充分发挥中俄蒙三国交界的区位优势和煤炭、石油等战略能源富集的资源优势，抓住用好东北振兴、西部开发等重要战略机遇期，以改革开放为动力，全力推进能源化工、进出口产品加工和农畜产品加工项目建设，加快具有地方和民族特色的城市化进程，积极推进新农村新牧区和生态建设，大兴文化旅游业，大力改善基础设施条件，切实加强精神文明和民主法治建设，高度关注民生，构建和谐社会，努力实现经济快速发展、生态环境更加良好，全力推动各项社会事业全面进步。

全市经济社会发展预期目标是：地区生产总值增长20％，达到480亿元，其中，全部工业增加值增长30％，达到136亿元；全社会固定资产投资增长50%以上，达到300亿元，其中，工业固定资产投资要达到或接近50%；财政收入增长20％，达到60亿元；城镇居民人均可支配收入增长14％，达到10320元；农牧民人均纯收入增长10.9％，达到4000元；城镇登记失业率控制在4.3％以内；人口自然增长率控制在5.17‰以内。

做好今年的各项工作，必须坚持以下五项原则：一是坚持科学发展的原则。大力转变经济增长方式，优化经济结构，加快能源、化工、旅游等优势特色产业发展，实现经济发展速度与质量同步提高。二是坚持生态优先的原则。加大生态保护和建设力度，合理开发利用可贵的自然资源，全力促进经济发展和生态保护协调共进，实现美丽与发展双赢。三是坚持关注民生的原则。切实关注广大人民群众的生产生活，将财力更多地向公共事业和民生方面倾斜，确保改革发展的成果更多地惠及全市各族人民。四是坚持开放带动的原则。充分利用“两种资源”，大力开拓“两个市场”，实现对俄蒙开放战略升级。五是坚持求真务实的原则。大兴务实之风，力戒形式主义，全力推动各项工作落实。

实现上述目标，我们要突出抓好以下十项重点工作：

（一）以推进新型工业化为核心，全力促进工业经济快速发展

认真研究国家宏观调控政策，高度重视大基地、大产业在国家产业规划中的地位，高度重视粗放型产业的深度转化，按照新型工业化的发展方向，加快煤炭、电力、化工等重点产业发展，优化市域新型工业体系结构，提高产业组合升级水平，全力扩张工业经济总量。实施市本级工业重点项目88个，工业固定资产投资达到140亿元，增长56.5％。规模以上工业增加值实现115亿元，增长30％。一要全力壮大优势特色产业。以优势资源转换为重点，以重点项目建设为支撑，在国家有保有压的政策环境中寻求机会，加快发展煤电、煤化工、石油、有色金属、绿色产品加工和进出口产品加工等优势产业。2007年，新增煤炭产量400万吨、电力装机125万千瓦，有色金属和黑金属采选日处理能力新增8000吨，鲜奶日处理能力新增1000吨，进口木材加工能力达到320万立方米。保证伊敏、宝日希勒两个露天矿具备年产千万吨的能力。全力推进扎煤公司灵东立井、鲁能五牧场河东区煤炭新开工项目的启动，争取扎罗木得新露天矿的启动。尽快完备核准手续，强力推动360万千瓦外送电源、深能源扎赉诺尔2×20万千瓦电源、海拉尔2×20万千瓦电源的尽快启动；努力推动乌奴克吐山铜钼矿、亚华乳业配方粉等项目开工建设。协调业主关系及项目审批条件，争取金新集团50万吨合成氨转80万吨尿素、上海重化40万吨甲醇、上海新大洲10万吨电石法PVC生产线、香江集团18万吨合成氨30万吨尿素等重大煤化工项目建设实现破题。同时，抓好扎煤灵泉矿300万吨升级改造、宝日希勒1000万吨露天矿、伊敏二期2×60万千瓦电源、谢尔塔拉铁锌矿、“七一”铅锌矿、雀巢公司日处理700吨鲜奶、洛娃乳业扩能、三发木材加工等续建项目建设。围绕出口机电产品生产加工逐步本地化和能源重化工产业延伸，积极引进制造业企业，实现电子产业的破题。积极支持满洲里发展进出口产品精深加工项目，支持森工集团通过资本运营发展林产品加工业和非林产业。二要全力支持十大企业发展。要高度重视森工集团、华能伊敏、大庆油田、神宝公司、鲁能大雁等十大企业在呼伦贝尔经济发展中的支撑作用。各旗市区、市直各部门、各园区要树立大企业兴呼伦贝尔兴的思想，与企业共谋发展大计，做到全天候、全方位、全身心、全过程为大企业服务。三要全力强化资源节约和环境保护。加大对战略资源的掌控力度，做到科学开发、合理利用。鼓励企业延伸产业链，发展低投入、低消耗、低排放、高效益的循环经济。做好工业企业的节能降耗工作。加强工业污染防治，坚持环境影响评价和“三同时”制度，切实抓好重点流域水污染、重点城市大气污染、重点工业污染源的监测和治理。保护好我们世代赖以生存发展的大草原、大森林、大水域。四要全力加强工业园区建设。抓好工业企业布局调整，新上、搬迁、改造的企业原则上都要按照规划入园，推动园区经济尽快形成规模；进一步明确园区管委会机构、职能、职责，建立考核激励机制。加强园区基础设施建设，积极争取上级资金支持，推动园区体制创新、技术创新，提高园区建设的档次和水平。力争园区工业增加值超过50亿元，销售收入实现100亿元以上。

（二）以推进现代农牧业发展为重点，扎实抓好社会主义新农村新牧区建设

高度重视“三农三牧”工作，牢牢抓住发展农村牧区生产力这个核心，大力推进新农村新牧区建设。一要积极推进现代农牧业建设。按照“传统产业优势化、支柱产业高效化、农牧业产业优质化、后备产业梯次化”的总体要求，努力提高农牧业的竞争力、产业层次和产出率。二要积极推进农牧民增收。建立农牧民增收长效机制，加大农村牧区基础设施和生态环境建设投入，改善农牧业生态环境，确保农牧业稳定增收，实现可持续发展；建立多元化的技术推广机制，进一步改革完善农牧业科技服务体系，加强农作物品种和畜禽良种体系建设，努力提高良种化水平和防检疫能力，确保农牧业提质增效。优化农牧业产业结构，大力发展畜牧业，突出发展农区畜牧业，全市牲畜总数达到1600万头（只），乳、肉产量分别达到135万吨和30万吨，通过产业结构调整促进农牧民增收。三要积极推进农牧业产业化进程。立足我市七大优势产业和十大绿色品牌，结合优势农畜产品产业集群建设和龙头企业加工的需要，大力发展特色、绿色、生态、安全农牧业，形成一批专业大户、专业村、专业乡镇，建立一批无公害、绿色农畜产品和出口产品生产基地。坚持“科学规划、积极引导、加强扶持”的原则，推进优势产品向优势企业集中，优势企业向优势区域汇集。在优势农畜产品区域内要重点扶持建设一批农畜产品加工园区，发挥区域规模效应。2007年，农牧业产业化百万元以上加工企业年销售收入达到63亿元以上，增长30%以上。认真落实各项惠农政策，大力发展各类经济合作组织和专业协会，畅通广大农牧民参与市场竞争的渠道。四要积极推进新农村新牧区建设。要按照“布局合理、以点带面、以线带片、点线结合、整体推进、体现特色”的思路，扎实推进新农村新牧区建设。整体推进2006年确定的90个规划补贴嘎查村，全面推广14个试点嘎查村经验，抓好不同类型的5个市级样板嘎查村建设。加大整治农村牧区村容脏乱差工作力度，改善农村牧区人居环境；提高农村牧区通车、通电、通广播、通电视覆盖面，改善农村牧区基础设施条件。

（三）以自治区成立60周年大庆为契机，大力发展富有地方和民族特色的文化旅游事业

围绕自治区成立60周年庆典活动，大力推进文化和旅游业发展。重点在四个方面实现新突破：一要在旅游与文化的结合上有新突破。把民族文化大市建设与旅游业发展有机结合，全力打造具有地方民族特色的旅游文化，赋予旅游项目以民族文化内涵。完成呼伦贝尔民族博物馆布展和成吉思汗广场改造工程，搞好以“三少”民族、俄蒙风情为主要风格的城市立面改造，进一步提升城市民族文化品位；做好额尔古纳蒙元文化博物馆和扎赉诺尔鲜卑古墓遗址博物馆前期规划，力争启动建设。推出五彩呼伦贝尔草原部落儿童合唱团。围绕自治区60周年大庆系列活动，认真做好呼伦贝尔文化旅游宣传促销工作。二要在旅游节庆活动档次上有新突破。充分发挥地域、民俗、历史、人文等优势，认真做好天堂草原大型实景演出规划。办好生态越野国际汽车拉力赛和赤塔-满洲里-海拉尔国际自行车拉力赛。开展国际草原那达慕、国际冰雪那达慕等系列活动，提升旅游节庆活动的文化内涵，打造旅游文化品牌。三要在旅游景区景点建设上实现新突破。改变旅游景区景点 “小、散、乱”的状况，按照“大规划、高起点、新立意”，整合市域内旅游资源，挖掘民族、历史文化底蕴，提高旅游景区景点的档次和品位。今年要重点开发建设呼伦贝尔民族风情园、海拉尔中俄蒙联合抗日胜利园、满洲里达赉湖小河口、额尔古纳西山风景区等重点项目。全力打造海拉尔-满洲里-额尔古纳“旅游金三角”、“两湖一湿地”、柴河-阿尔山、牙克石冬季旅游中心等精品旅游线路。四要在旅游基础设施建设上有新突破。承接旅游业的大好发展来势，加大旅游基础设施建设力度。提高公路、铁路、民航的运输能力，改善旅游交通条件；加大海、满、牙、扎等重点旅游城市及旗市中心城镇的宾馆、酒店、娱乐、购物场所建设力度。力争全年接待游客480万人次，旅游业总收入达到65亿元,增长21%。

（四）以提高可持续发展能力为目标，加快生态环境建设进程

以生态市建设为契机，全面加强生态环境建设。在具体措施上，做到三个不动摇：一是实施退耕和禁牧休牧的决心不动摇。全年完成退牧还草320万亩，退耕100万亩，完成草地围栏320万亩，改良草地60万亩，人工补播、飞播牧草6万亩，人工种草80万亩。实施禁牧500万亩、休牧4000万亩、划区轮牧175万亩，签订草畜平衡责任书的草场面积达到9700万亩以上。二是加大林业生态建设力度不动摇。继续实施好“天保工程”，通过采取划区保护、禁伐、限伐等措施，加强森林资源管护。继续加大项目争取力度，组织开展好重点公益林保护、退耕还林、三北防护林、防沙治沙等工程项目建设。加大社会化造林和全民义务植树力度，进一步提高森林覆盖率。积极支持中国荒漠化治理基金会呼伦贝尔草原沙化治理项目。三是坚持科学开发与合理利用资源的思路不动摇。停止一切导致生态功能退化的开发和其他人为破坏活动，切实保护好呼伦贝尔森林、湿地、草原的生态功能。今年要在适当时间召开全市生态示范区建设表彰大会暨生态市建设动员大会，在全市掀起生态保护和建设的高潮。

（五）以推进城市化进程为引领，加快各项基础设施建设步伐

按照“优化布局、提升功能、适度超前”的原则，切实加强全市基础设施建设，增强对经济发展的支撑能力。城市建设：一要加快首府城市海拉尔的建设步伐。拉开城市框架，扩大城区规模，提高城市品位，完善城市功能，把海拉尔打造成适宜人居、适宜创业的现代草原首府城市，打造成全市政治、文化、商贸中心，打造成全市经济发展核心区。今年要在自治区60周年庆典前，完成沿街372栋楼房立面改造工程；抓好易必斯酒店、炎皇世纪国贸商厦和东出口道路拓宽改造项目建设，提升首府城市品位。二要加快边境口岸城市满洲里的建设步伐。充分发挥满洲里在全市对俄蒙开放中的龙头作用，积极支持满洲里城市建设，切实把满洲里建设成国际性综合口岸名城。重点启动扎区、东湖区新城市功能区建设，完成12条街道两侧重点楼房外立面欧式改造，努力营造浓郁的欧式古典建筑风格。三要加快各旗市中心城镇建设步伐。突出边境城镇、林区城镇、农区城镇、牧区城镇特色，重点抓好给排水、集中供热、垃圾处理、污水处理、市政道路、园林绿化等城市基础设施建设，完善城市功能，增强聚集生产要素和辐射带动能力。四要加快呼伦贝尔新城区建设步伐。今年要进一步完善西区、启动东区，完成16条20公里的道路建设，启动广场、公园、热电厂、供水等工程建设以及新城区绿化工程。加大入驻新区力度，尽快把新区人气旺起来。交通建设：确保海拉尔-满洲里一级公路竣工通车，开工大杨树-宜里、蘑菇气-阿尔山等公路建设项目，争取开工建设甘南界-牙克石高速公路项目。加快铁路主要干线的后方通道及延伸建设，加快滨洲铁路海满段复线和“两伊”铁路建设步伐，抓好满洲里-阿尔山、莫尔道嘎-室韦、海拉尔-黑山头、扎兰屯-阿荣旗-莫旗地方铁路项目的前期立项核准和融资工作。组织开工民航海拉尔机场飞行区项目，完成满洲里机场改（扩）建项目。水利建设：开工建设红花尔基水库工程和嫩江干流尼尔基-博荣-汉古尔河及东西诺敏河防洪工程。做好扎罗木得、毕拉河口水利枢纽及尼尔基水库下游灌区的前期工作。抓好呼伦湖水资源配置及水环境治理工程前期和资金落实工作，尽快开工建设。进一步完善农田草牧场水利基本建设投入机制，加快农田水利建设步伐，提高农牧业综合生产能力。继续加大安全饮水工程建设力度，让更多的群众喝上安全水、放心水。

（六）以提高经济社会发展活力为方向，加大改革开放力度

全面深化各项改革。农垦企业改革要坚持政企分开的原则，以集团化、股份化、产业化为目标，加快改革步伐，年内要取得实质性进展。地方林业改革要进一步理顺管理体制，加快经营性企业的市场化进程。煤炭企业改革要加大优化整合力度，实现投资主体多元化。深化农牧业基层服务体系改革，加强农牧业推广体系建设，加快种子管理体制改革，尽快实现政企分开、政事分开。深化财政体制改革，加强税收征管，培植税源，挖潜增收，提高财政收入质量。重视和加强审计工作，对重点领域、重点项目、重点资金进行重点审计监督，严肃查处违法违纪行为，规范财经秩序。

全面抓好招商引资。大力优化投资发展环境，着力打造具有政策、资源、产业优势的引资“洼地”，加大“走出去、请进来”力度，多渠道引进国内外知名大企业、大集团入驻呼伦贝尔，增强地区经济发展活力。重点抓好与东北三省的产业对接，在能源基地建设、绿色食品、旅游业等方面加强合作，争取构建“哈大齐呼经济协作区”，借东北振兴之力加快发展。加强与环渤海经济区、“长三角”、“珠三角”等发达地区的联系和沟通，积极承接产业转移，杜绝落后技术、污染企业进入。全年引进市外（国内）资金220亿元，增长45%。 全面扩大对外开放。以俄罗斯“中国年”活动为契机，提高对外开放水平。进一步完善“大通关”体系，抓好满洲里互市贸易区、进出口产品加工区建设。实施黑山头、室韦口岸联检楼建设项目。做好阿日哈沙特中蒙跨境铁路、黑山头口岸项目前期论证工作，争取实现阿日哈沙特口岸国际性常年开关，额布都格口岸正式对外开放并常年临时开关。围绕境外资源开发，加快口岸经济发展。继续扶持新鑫矿业有限公司蒙古国乌兰铅锌矿开采、大庆油田蒙古国塔木察格地区石油开发等项目。2007年，对外贸易进出口总额实现23亿美元，口岸过货量达到2400万吨。

（七）以培育新经济增长点为目的，加快第三产业和非公有制经济发展

坚持以市场体系建设为重点，全面提升第三产业发展水平。努力促进商贸流通、运输邮政、餐饮等传统服务业升级改造，积极培育物流、会展、金融、信息、中介等现代服务业。积极推动房地产业健康持续发展，适度扩大经济适用住房的建设规模，全面实施廉租住房制度。加强社会信用体系建设，建立健全中小企业投资担保体系。积极开展与国家开发银行、信托机构和市内金融机构的融资合作，努力拓宽融资渠道。继续推进农村信用社改革，创新资本运作方式，组建农村合作银行，提高为“三农三牧”服务的水平。

坚持以扩大规模和提升档次为重点，大力发展非公有制经济。认真贯彻落实我市《关于鼓励支持和引导个体私营等非公有制经济发展的具体实施意见》和《发展个体私营等非公有制经济各旗市区考核办法（试行）》。通过放宽准入，扩大非公有制经济总量；通过招商引资，大量引进外地非公有制企业；通过启动民间资本，积极培育本土非公有制企业发展壮大；通过政策导向作用，鼓励非公有制企业发展优势特色产业，参与新农村新牧区建设、国有企业改革和基础设施建设。建立健全非公有制经济工作机构，形成全力扶持非公有制经济加快发展的强大合力，推进非公有制经济总量迅速扩张。力争非公有制经济在全市经济总量中的比重达到37%以上，投资在全社会固定资产投资中的比重达到20％以上，销售收入超亿元的大型民营企业达到30家以上。

（八）以促进和谐社会建设为根本，加快各项社会事业发展

进一步深化科技体制改革，积极争取国家专项资金支持，抓好科技创新能力建设和科技成果转化，推进科技示范园区、特色产业化基地建设，全面实施“科技特派员行动”；建立健全科技人才培养引进机制，营造适合科技人才建功立业的良好环境，培养本土人才，引进域外人才，留住现有人才，加快实施科技兴市战略步伐。加快各级各类教育发展，重视农村教育，优先发展民族教育，大力发展职业教育，稳步推进中考改革，千方百计满足人民群众的教育需求；抓好农村牧区“两免一补”和“民族学校少数民族贫困寄宿生助学金”的政策落实，巩固牧区“两主一公”的办学模式。大力改善农村牧区卫生基础设施条件，全面实施新型农村牧区合作医疗，进一步做好乙肝、结核病、流行性出血热等重点传染病和地方病的防治工作，加强城市社区卫生服务体系建设，提高蒙中医药开发利用水平，努力保证人民群众公平享有基本卫生保健。加强食品药品安全监管，保障人民群众饮食用药安全。全面加强人口和计划生育工作，继续稳定低生育水平。大力推进文化大市建设，积极打造民族文化品牌，培养民族文化人才。以自治区成立60周年大庆文艺调演为契机，继续推进民族艺术精品战略，大力发展文艺事业。抓好12个乡镇的草原万里健身工程。积极推进广播电视数字化，实施广播电视村村通和无线覆盖工程，广播电视人口覆盖率达到88%。抓好就业和再就业工作，全年城镇新增就业2.85万人，城镇登记失业率控制在4.3%以内，“就业困难对象”实现再就业6500人，农村劳动力外出就业3.2万人。做好养老保险市级统筹工作。加大社会保险参保扩面力度，开展农村牧区养老保险和城镇居民医疗保险试点工作，积极推进森工、地方煤炭企业参加工伤保险，促进“平安计划”的实施。执行地区最低工资标准，提高企业退休职工养老金标准，兑现公务员工资制度改革政策。城镇居民最低生活保障标准提高20元，达到143元；农村牧区低保人数达到3.5万人，特困农牧民低保金做到按时足额发放。积极争取国家“兴边富民”重点县项目，全面落实国家扶持人口较少民族发展“十一五”规划，着力改善鄂温克族、鄂伦春族、达斡尔族和俄罗斯族的生产生活条件，加快少数民族群众和少数民族聚居区经济社会发展。实施人才强市战略，加强人事人才工作。组织开展好全国第二次农牧业普查工作。认真做好扶贫、人防、气象、地震、档案、宗教、通讯、邮政、保险和对外联络等工作，为全市经济社会和谐发展提供保障。

（九）以创建“安全稳定呼伦贝尔”为保障，全面加强精神文明和民主法制建设

加强精神文明建设。广泛深入开展社会公德、职业道德、家庭美德教育，加强青少年思想道德建设，进一步提高市民素质，形成健康文明的生活方式、平等宽容的人际关系、知荣明耻的社会风尚。加强政务、商务、社会诚信建设，增强全社会诚实守信意识。把和谐社区、和谐家庭等和谐创建活动同群众性精神文明创建活动结合起来，深入开展城乡社会志愿服务，继续开展文明城、双拥模范城等争创活动。力争实现全国双拥模范城“两连冠”目标。

加强民主法制建设。提高政府依法行政水平，确保人民赋予的权力更好地为人民服务。自觉接受人大依法监督、政协民主监督、新闻舆论监督和社会公众监督，认真办理人大代表建议、议案和政协委员提案。改革和完善决策机制，严格规范各级政府的决策规则和程序，健全重大决策科学论证和专家咨询制度，提高科学决策水平。高度重视人民群众来信来访工作，进一步健全科学有效的诉求表达、矛盾纠纷调处和信访工作问责机制。建立完善群体性事件预警、应急、处置机制，提高保障公共安全和处置突发事件的能力。

加强维护稳定工作。继续开展创建“安全稳定呼伦贝尔”活动，加强社会治安综合治理，深入推进公安机关“三基”工程建设，依法严厉打击各种刑事犯罪和敌对势力的分裂破坏活动。深入开展法制教育，落实依法治市和“五五”普法规划，做好法律服务和援助工作。建立健全国防动员体系，支持国防重点工程建设，继续巩固边防一体化建设成果。认真落实各项安全生产责任和监管措施，切实抓好煤矿、交通、森林草原防火、城镇消防和高危行业的安全生产，确保全年不发生重特大事故。

（十）以人为本，全力为民办十件实事

今年，市委、市政府决定为人民办10件实事。一是安置原国有企业3300名“4050”人员再就业，确保每个零就业家庭有1人就业。二是解决国有关闭破产企业退休人员、各类困难企业人员和农牧民工参加住院医疗保险问题，解决被征地农（牧）转非人员参加医疗保险问题。三是全面推行新型农村牧区合作医疗制度，100%覆盖全市农牧业人口。四是对乙型肝炎、流行性出血热高发地区的重点人群实施免费接种疫苗。乙肝疫苗接种人数17.3万人，流行性出血热疫苗接种人数5.6万人。五是资助200名农村牧区贫困家庭子女免费接受中等职业教育。六是扩大中小学贫困家庭寄宿生生活费补助范围，在2006年补助21000人的基础上增加10个百分点，达到23100人。七是在2006年少数民族群众危房改造的基础上，以“三少”民族为重点，少数民族群众危草房改造达到6000户。八是新增10部调频广播发射机，使市本级广播节目的覆盖率达到65%以上。九是在海拉尔区建1所为老年人服务的多功能文体活动中心。十是新建10所农村敬老院，新建1所市直光荣院，改善老年人的养老条件。全市孤儿生活补助标准每人每月提高30元。

三、建设人民满意政府

要从提高政府的“执行力、创新力、凝聚力、公信力”入手，努力形成集中精力“想干事”、开拓创新“会干事”、团结奋斗“干成事”的良好氛围，建设让人民满意的政府。

第一，切实提高政府的执行力。要切实强化大局意识，确保政令畅通。牢固树立全市“一盘棋”的思想，认真贯彻落实上级的决策部署，切实把市委、政府确定的各项工作任务落到实处。全面抓好公务员队伍建设，进一步提高各级干部的综合素质，加强工作实践，培育团队精神，形成整体合力。大力营造真抓实干的良好氛围，进一步解放思想，以攻坚克难的勇气，对待前进中的矛盾和问题；以开拓创新的锐气，对待已有的成绩和经验；以积极应对的豪气，对待各种风浪和挑战，形成一级做给一级看、一级带着一级干的局面。要严明奖惩，严肃组织纪律和工作纪律，进一步加大监督管理力度，建立问责制度，开展绩效评估，形成一级抓一级、层层抓落实的工作推进机制，全面提高政府的执行能力。

第二，切实提高政府的创新力。要在继承的基础上，实现观念、机制、方法创新。一是观念要创新。面对日新月异的发展形势，政府的各级领导干部，一定要跟上时代发展步伐，紧扣和谐社会建设的主旋律，以实现呼伦贝尔在科学发展中崛起为指针，统揽政府工作全局，更新发展观，更新政绩观，更新荣辱观，切实把思想统一到科学发展的观念上来。二是机制要创新。要抓住事关全市改革发展稳定大局的重点、难点工作，组成分线推进组，加大督促、检查、协调、服务力度；充分利用高科技手段，以电子政务信息化为平台，依托互联网资源，加强政务办公系统、项目审批管理系统、农村综合信息服务系统的建设，提高行政效能。三是方法要创新。在工作方法上，要把握好“多、活、硬”三个方面：多，就是方法要多。各级领导干部要增强应对复杂局面的能力，增强工作的预见性、前瞻性，未雨绸缪，做好克服困难、解决矛盾、促进工作的多项预案，推动各项工作卓有成效地开展。活，就是方式要活。要顺应国家产业政策、国际经济环境的变化，灵活掌握推进工作的方法，尤其是在抓项目谋划、核准、招商等方面一定要灵活机动，适应各种不同的变化，以活化的工作方法应对变化的环境。硬，就是作风要硬。各级领导干部一定要敢于负责，有利于呼伦贝尔经济社会发展、有利于提高人民生活水平、有利于维护地区稳定的事项，一定要敢抓敢管，敢于碰硬，敢于推进。

第三，切实提高政府的凝聚力。一要以加快发展凝聚人心。充分认识和把握现有的大好来势，充分发挥资源、区位、政策、人气等诸多优势，激发全市各级干部、各族人民求发展、盼富裕的强烈愿望，在全市形成心往一处想、劲往一处使、共谋发展的良好氛围。二要以团结协作凝聚人心。政府班子及组成部门一定要讲团结、讲协作、讲统一，加强沟通联系，保证政府系统纵向横向步调一致、运转协调。三要以关注民生凝聚人心。我们一定要清醒地认识到我市城乡收入水平相对较低、贫困人口较多这个现实，在工作摆布、财力安排、政策导向上，积极向惠民事业倾斜，以人为本，高度关注各族人民的生产生活，通过各级、各部门的共同努力，切实解决好社会、民生、弱势群体等问题，在公众中树立良好的政府形象。

第四，切实提高政府的公信力。公信力是政府的影响力与号召力，体现了政府工作的权威性、民主程度和法治建设程度。一要依法行政。依法行政是诚信社会的重要标志，也是政府及各部门必须遵守的准则。各级、各部门的领导干部要加强对相关法律知识的学习，增强法治观念，提高依法行政能力。二要强化服务。牢固树立“管理就是服务、服务就是义务”的观念，以服务为天职，以优化环境为己任，切实把“服务至上”的理念贯穿到各项工作之中。切实为旗市区、大企业、民营经济做好服务，千方百计化解各种矛盾，让基层的同志轻装上阵，全身心地抓好工作落实。三要廉洁自律。认真贯彻落实国务院第五次廉政建设工作会议精神，加强政风建设，完善反腐倡廉制度，推行政务公开，开展治理商业贿赂专项工作，着力解决损害群众利益的突出问题。各级领导干部要时刻自重、自省、自警、自励，在思想上筑牢拒腐防变的防线，严格执行党风廉政建设的各项规定，自觉接受党内外的监督，不仅要管好自己，也要管好亲属和身边的工作人员。做到既勤政又廉政，既干事又干净，做到不当庸官、不当懒官、不当昏官、不当贪官。

各位代表，目标已明确，蓝图已绘就；机遇催人奋进，使命崇高神圣。让我们在自治区党委、政府和市委的正确领导下，借助自治区成立60周年、呼伦贝尔撤盟设市5周年的东风，紧紧依靠全市各族人民，团结一心, 扎实工作，拼搏进取，开拓创新，为构建和谐呼伦贝尔、实现呼伦贝尔在科学发展中崛起而努力奋斗！
